# Associations between Anxiety, Depression, Antidepressant Medication, Obesity and Weight Gain among Canadian Women

**DOI:** 10.1371/journal.pone.0099780

**Published:** 2014-06-16

**Authors:** Anne Grundy, Michelle Cotterchio, Victoria A. Kirsh, Nancy Kreiger

**Affiliations:** 1 Prevention and Cancer Control, Cancer Care Ontario, Toronto, Ontario, Canada; 2 Dalla Lana School of Public Health, University of Toronto, Toronto, Ontario, Canada; 3 Department of Nutritional Sciences, University of Toronto, Toronto, Ontario, Canada; Federal University of Rio de Janeiro, Brazil

## Abstract

**Purpose:**

Some mental illnesses have been suggested to be associated with obesity, although results are somewhat inconsistent and research has focused mainly on depression.

**Methods:**

Associations between anxiety, depression, medications for these illnesses, and obesity were investigated cross-sectionally among women aged 25–74 (n = 3004) who participated as population controls in a cancer case-control study. Participants self-reported information on anxiety, depression, height, current weight and weight at age 25.

**Results:**

No association was observed between either anxiety or depression and either current overweight or obesity status. However, depressed women taking antidepressants were more likely to be obese [OR = 1.71 (95%CI  =  1.16–2.52) daily antidepressant use; OR = 1.89 (95%CI = 1.21–2.96) ever tricyclic antidepressant use]. In the full study sample consistent positive associations between anxiety, depression and obesity among women with a history of antidepressant use, and generally negative associations among women without, were suggested. Finally, weight gain was associated with history of anxiety [5–19 kg OR = 1.29 (95% CI = 1.06–1.57); ≥20 kg OR = 1.43 (95% CI = 1.08–1.88)] and depression [≥20 kg OR = 1.28 (95% CI = 0.99–1.65)].

**Conclusions:**

These results suggest depression and anxiety may be associated with weight gain and antidepressant use may be associated with obesity.

## Introduction

Obesity is a risk factor for several health conditions including cardiovascular disease, diabetes and some types of cancer [1]. In addition to well-established determinants like diet and exercise, there is a need to investigate alternative risk factors of this multifactorial disease [2]–[4] and some mental illnesses have been identified as potential contributors [5]. Some epidemiologic evidence has suggested a relationship between several mental illnesses and obesity [6]–[26], although other studies have not found similar associations [27]–[31].

Cross-sectional studies have focused mainly on depression and although several studies suggested a positive association [6]–[12], others observed either inverse or null relationships [27], [28]. Findings for other mental illnesses are similar, with links between obesity and both mood and anxiety disorders [13]–[17]. Most prospective studies have focused on relationships between anxiety or depression in adolescence and obesity in either later adolescence [24], [30]–[32] or early adulthood [18]–[22] with only a few examining adult depression and later obesity [12], [25], [26], [33]. In studies of adulthood, follow up periods have generally been short and while some have demonstrated relationships between depression and later obesity [25], [33], others have shown only associations at baseline [12], [26]. Most prospective work has focused on depression [19]–[22], [25], [30]–[33], with few studies considering other illnesses like anxiety [18], [24], [26]. One recent review indicated only 8/15 prospective studies identified depression as a significant predictor of obesity [5], while another meta-analysis detected reciprocal relationships, where the association between depression and obesity development was marginally stronger [34].

Several biological mechanisms have been postulated to explain relationships between mental illness and obesity. These include medication use (specifically antidepressants), impaired sleep quality, overeating and physical inactivity [5]. Regarding antidepressant use, tricyclic antidepressants (TCAs) and monoamine oxidase inhibitors (MAOIs) have been associated with greater weight gain than other classes [35], [36]. To our knowledge, only two studies have specifically considered the role of medication use in relationships of depression with obesity. However, both considered only major depression and only current use of antidepressant medications was evaluated [37], [38].

Thus, while some research has suggested an association between depression and obesity, results are not entirely consistent [5] and few studies have directly examined these relationships in adulthood [12], [25], [26], [33]. Further, the body of evidence concerning mental illnesses beyond depression is smaller [13]–[17] and additional work including conditions like anxiety is needed. The purpose of this study was to examine associations of history of depression and anxiety with current overweight and obesity status, as well as weight gain. We also considered the impact of antidepressant medication use on these relationships.

## Methods

### Ethics Statement

Ethics approval for this analysis was provided by the University of Toronto Research Ethics Board.

### Study Sample

Women included in this study were population controls from the Women's Health Study, a breast cancer case-control study, details of which have been previously described [39], [40]. Briefly, controls were women recruited from the 1996 Ontario Ministry of Finance assessment roll, which includes both home owners and tenants. Controls were randomly selected from these rolls and frequency matched in 5-year age groups to breast cancer cases. The overall response rate for controls was 61% (3062/5001), of whom 3004 were ages 25–74 years, and were included in this cross-sectional analysis. Women who participated in the study were asked to complete self-administered questionnaire, which collected demographic information, reproductive history, family and personal medical history including history of mental illness, medication use, and lifestyle characteristics including smoking and physical activity.

### Anxiety, Depression and Obesity Assessment

The main mental illness variables used in this analysis were: anxiety, depression and anxiety and/or depression. History of anxiety and depression was self-reported by participants, where women were asked to indicate whether they had ever suffered from the condition and if yes, the age at which they first experienced the problem. The Women's Health Study used diagnostic categories from DSM-I [41], the Structured Clinical Interview for DSM-III-R [42] and the National Institute of Mental Health Diagnostic Interview Schedule [43] in the development of questions regarding history of mental illness.

Overweight and obesity were assessed through self-report in the study questionnaire: women were asked to report their height and current weight (three years prior to study participation). Individuals were categorized as overweight if their Body Mass Index (BMI, kg/m^2^) was ≥25 and <30 and as obese if their BMI was ≥ 30. Weight at age 25 years was also reported and weight gain (in kg) between age 25 and current weight was also considered. Current BMI was missing for 62 (2.1%) women and weight gain for 87 (2.9%).

### Statistical analysis

Descriptive characteristics were presented using means and standard deviations for continuous variables and percentages for categorical variables. Differences between normal, overweight and obese groups were assessed using analysis of variance for continuous variables and chi-square tests for categorical variables.

Depression and anxiety were the primary exposure variables and measures of obesity the outcome. Multivariate polytomous logistic regression was used to calculate odds ratios (ORs) and 95% confidence intervals for the associations of each mental illness category with overweight and obesity. Similar models also examined the influence of age at first incidence of anxiety and depression on overweight and obesity. Relationships for anxiety, depression and anxiety and/or depression with weight gain in one of three categories (0–4 kg, 5–19 kg and ≥ 20 kg) were also examined using polytomous logistic regression, with the 0–4 kg category as the referent. Associations between ever having used antidepressant medications, both in general and specifically TCAs, MAOIs and selective serotonin reuptake inhibitors (SSRIs) with overweight and obesity for all study participants, as well as only among those who reported a history of depression, were examined. Relationships of anxiety and depression with overweight and obesity were examined stratified by ever use of antidepressant medications.

Potential confounders were variables that might be associated with either mental illness or obesity and those considered in the multivariate model were age, marital status, education, household income (previous three as measures of socio-economic status), menopausal status, smoking, alcohol consumption and physical activity. Women were considered post-menopausal if they had stopped menstruating three or more years prior to study participation (time of BMI measurement) or if menstruation had stopped and they were 58 years or older. To characterize physical activity patterns, women were asked to report the number of times per month they engaged in at least 20 minutes of moderate and strenuous physical activities during four lifetime periods (teens, 30s, 50s and 3 years ago). A physical activity index was created by summing these values and quartiles of the physical activity index were used, with higher values for those more active over their lifetime. Age (continuous) was forced into the model and all other confounders were assessed using an all-possible-models manual backwards selection procedure [44], where variables that changed ORs by >10% were retained in the final model. All analyses were conducted using SAS, Version 9.2 (Cary, NC).

## Results

Characteristics of normal weight participants in comparison to overweight and obese women are shown in Table 1. No associations were observed for history of anxiety, depression or anxiety and depression combined with either overweight or obesity (Table 2). With age forced into the model, no other potential confounders changed the observed odds ratios by greater than 10% and thus were not included in the final multivariate model. When the overweight and obese groups were pooled to increase power, results were similar, where no associations were observed in logistic regression models adjusted for age (data not shown).

**Table 1:** Descriptive Characteristics of Study Sample by Categories of Current BMI.

	FULL POPULATION			
	Normal (n = 1465)	Overweight (n = 936)	Obese (n = 541)	*P* value
	*N/Mean (%/SD)*	*N/Mean (%/SD)*	*N/Mean (%/SD)*	
Age	54.9 (11.5)	57.4 (10.5)	57.7 (10.3)	<0.0001
Marital Status				0.20
Married/Common Law	1091 (74%)	732 (78%)	405 (75%)	
Divorced/Separated	145 (10%)	74 (8%)	39 (7%)	
Widowed	159 (11%)	98 (10%)	68 (13%)	
Never Married	66 (5%)	29 (3%)	27 (5%)	
Missing	4 (0.3%)	3 (0.3%)	2 (0.4%)	
Education				<0.0001
No Formal Education	7 (0.5%)	6 (1%)	8 (1%)	
Elementary or less	139 (9%)	140 (15%)	99 (18%)	
High School or less	642 (44%)	436 (47%)	255 (47%)	
Postsecondary	669 (46%)	349 (37%)	177 (33%)	
Missing	8 (1%)	5 (1%)	2 (0.4%)	
Household Income				0.002
<$10,000	35 (2%)	27 (3%)	16 (3%)	
$10,000–$19,999	93 (6%)	75 (8%)	50 (9%)	
$20,000–$29,999	167 (11%)	127 (14%)	78 (14%)	
$30,000–$49,999	297 (20%)	217 (23%)	123 (23%)	
$50,000–$100,000	463 (32%)	240 (26%)	126 (23%)	
>$100,000	119 (8%)	57 (6%)	25 (5%)	
Not stated	251 (17%)	168 (18%)	107 (20%)	
Missing	40 (3%)	25 (3%)	16 (3%)	
Menopausal Status				
Premenopausal	670 (46%)	311 (33%)	178 (33%)	<0.0001
Postmenopausal	771 (53%)	613 (65%)	358 (66%)	
Missing	24 (2%)	12 (1%)	5 (1%)	
**Lifestyle Characteristics**				
Smoking Status				<0.0001
Current Smoker	335 (23%)	162 (17%)	81 (15%)	
Ex-Smoker	389 (27%)	249 (27%)	187 (35%)	
Never Smoker	722 (49%)	515 (55%)	268 (50%)	
Missing	19 (1%)	10 (1%)	5 (1%)	
Alcohol Consumption (# drinks per week)				<0.0001
None	319 (22%)	260 (28%)	176 (33%)	
>0–1	378 (26%)	236 (25%)	166 (31%)	
>1–2.5	339 (23%)	198 (21%)	98 (18%)	
>2.5 (max 21)	407 (28%)	219 (23%)	88 (16%)	
Missing	22 (2%)	23 (2%)	13 (2%)	
Physical Activity Index				<0.0001
0–45.3	287 (20%)	228 (24%)	127 (23%)	
>45.3–70	296 (20%)	202 (22%)	143 (26%)	
>70–98	326 (22%)	214 (23%)	123 (23%)	
>98 (max 160)	367 (25%)	196 (21%)	85 (16%)	
Missing	189 (13%)	96 (10%)	63 (12%)	

a. 62 individuals missing obesity status, 40 of these were postmeonopausal and 19 premenopausal (3 missing menopausal status and obesity)

**Table 2:** Association between Anxiety, Depression and Current BMI.

	Normal (n = 1465)	Overweight (n = 936)		Obese (n = 541)		*P* value (trend)
	*N*	*N*	*OR (95% CI)^a^*	*N*	*Odds Ratio^a^*	
**Ever Diagnosis:**						
Anxiety						
Yes	335	215	1.05 (0.86–1.28)	117	0.98 (0.77 – 1.25)	0.62
No	1111	705		411		
Depression						
Yes	425	256	0.96 (0.80 – 1.16)	144	0.94 (0.75 – 1.17)	0.82
No	1022	655		386		
Depression/Anxiety						
Yes	540	331	0.98 (0.82 – 1.04)	180	0.90 (0.73 – 1.11)	0.48
No	917	601		356		
**Age at Diagnosis:**						
Anxiety						
Never	1111	705		300		
≤ 18	81	50	1.03 (0.72 – 1.49)	25	1.10 (0.72 – 1.70)	0.79
19 – 30	84	60	1.20 (0.85 – 1.70)	27	0.89 (0.57 – 1.41)	0.22
31 – 40	67	47	1.18 (0.80 – 1.74)	23	1.12 (0.70 – 1.79)	0.84
≥ 41	103	58	0.86 (0.61 – 1.20)	28	0.86 (0.58 – 1.30)	0.98
Depression						
Never	1022	665		273		
≤ 18	87	61	1.17 (0.83 – 1.65)	20	0.73 (0.45 – 1.18)	0.07
19 – 30	105	72	1.16 (0.84 – 1.60)	45	1.23 (0.85 – 1.79)	0.78
31 – 40	96	47	0.82 (0.57 – 1.18)	16	0.75 (0.47 – 1.19)	0.74
≥ 41	137	76	0.82 (0.61 – 1.10)	48	0.98 (0.70 – 1.38)	0.34

a. Model adjusted for age.

The association between weight gain since age 25 and mental illness revealed elevated ORs for anxiety for both the 5 – 19 kg and ≥20 kg weight gain categories (Table 3). Further, an increased risk in the highest category of weight gain was observed for women with a history of depression and for anxiety and depression combined, although these were of marginal statistical significance. To assess the impact of potential difficulties in recall of weight at age 25 for older women, analysis of the relationship with weight gain for both depression and anxiety was stratified by age at age 50 years. ORs among women age <50 were similar to those of the overall analysis, while those among women age ≥50 were closer to 1.0 (Table S1), suggesting there may have been differences in the ability to recall past weight between older and younger women.

**Table 3:** Association between Anxiety, Depression and Weight Gain since age 25.

Mental Illness	0 – 4 kg (n = 1012)	5 – 19 kg (n = 1493)		>20 kg (n = 412)		*P* value (trend)
	*N*	*N*	*OR (95% CI)^a^*	*N*	*OR (95% CI)^a^*	
Ever Diagnosis:						
Anxiety						
Yes	209	357	1.29 (1.06 – 1.57)	103	1.43 (1.08 – 1.88)	0.43
No	793	1108		300		
Depression						
Yes	288	406	0.99 (0.83 – 1.19)	129	1.28 (0.99 – 1.65)	0.04
No	710	1066		273		
Depression/Anxiety						
Yes	855	537	1.11 (0.93 – 1.31)	157	1.27 (1.00 – 1.61)	0.25
No	652	946		252		
Age at First Diagnosis:						
Anxiety						
Never	793	1108		411		
≤18	56	82	1.11 (0.76 – 1.63)	31	1.31 (0.78 – 2.19)	0.59
19 – 30	50	94	1.76 (1.16 – 2.66)	26	1.96 (1.15 – 3.35)	0.88
31 - 40	47	70	1.33 (0.86 – 2.05)	26	1.68 (0.96 – 2.94)	0.49
≥41	56	111	1.33 (0.92 – 1.93)	34	1.22 (0.74 – 2.01)	0.48
Depression						
Never	710	1066		386		
≤18	61	89	1.09 (0.77 – 1.54)	22	1.01 (0.59 – 1.72)	0.77
19 – 30	69	108	1.19 (0.86 – 1.64)	44	2.09 (1.38 – 3.14)	0.004
31 - 40	67	85	0.93 (0.66 – 1.31)	25	0.73 (0.41 – 1.29)	0.38
≥41	91	124	0.86 (0.65 – 1.15)		1.28 (0.88 – 1.87)	0.03

a. Model adjusted for age.

Age at first incidence of anxiety and depression was evaluated in one of four time windows: ≤18, 19 – 30, 31 – 40 and >40. No associations were observed for overweight or obesity with either anxiety or depression during any of the four time windows (Table 2). Anxiety first experienced between 19 and 30 years of age seemed to have the strongest association with weight gain, and depression first experienced in the same age range produced the largest OR for the ≥20 kg weight gain category (Table 3).

In the full study sample, while ORs for relationships between antidepressant use and overweight/obesity were generally elevated in the obese group, all confidence intervals included 1.0 (Table 4). However, when this analysis was restricted to women with depression, those taking antidepressants (both general use and use of TCAs specifically) were more likely to be obese (Table 4). No associations with either overweight or obesity were observed for use of MAOIs or SSRIs. For MAOIs, the lack of association with obesity may have been due to the small number of individuals who reported using this type of medication, as the confidence interval was quite wide (Table 4). Further, when associations of depression and anxiety with overweight and obesity were stratified by medication use, estimated ORs were generally above 1.0 in the group that had used antidepressant medications and below 1.0 in the group that had not (Table 5). However, most confidence intervals associated with these estimates included 1.0 and, specifically in the group that used antidepressant medication, the number of women who did not report experiencing anxiety or depression was quite small, such that the pattern of elevated risk in the medication group should be interpreted cautiously.

**Table 4:** Antidepressant Medication use and Current Obesity.

	ALL PARTICIPANTS						PARTICIPANTS REPORTING HISTORY OF DEPRESSION ONLY^a^					
	Normal (n = 1433)	Overweight (n = 921)		Obese (n = 525)		p-value (trend)	Normal (n = 425)	Overweight (n = 256)		Obese (n = 144)		p-value (trend)
	*N*	*N*	*OR (95%CI)^b^*	*N*	*OR (95%CI)^b^*		*N*	*N*	*OR (95%CI)^b^*	*N*	*OR (95%CI)^b^*	
Daily Medication to treat depression												
Yes	183	115	0.99 (0.77–1.27)	77	1.19 (0.89–1.57)	0.24	161	104	1.14 (0.83–1.57)	73	1.71 (1.16–2.52)	0.05
No	1250	806		448			256	144		67		
Ever Use of TCAs												
Yes	88	62	1.07 (0.77–1.50)	46	1.43 (0.98–2.07)	0.16	71	51	1.23 (0.82–1.84)	40	1.89 (1.21–2.96)	0.08
No	1346	859		479			347	197		100		
Ever Use of MAOIs												
Yes	5	2	0.59 (0.11–3.08)	3	1.57 (0.37–6.61)	0.29	5	2	0.64 (0.12–3.33)	3	1.70 (0.40–7.23)	0.29
No	1428	919		522			412	246		137		
Ever Use of SSRIs												
Yes	95	45	0.79 (0.55–1.14)	32	1.01 (0.66–1.53)	0.31	89	40	0.74 (0.49–1.12)	31	1.12 (0.70–1.79)	0.12
No	1338	876		493			328	208		109		

a. 14 individuals missing obesity status.

b. Model adjusted for age.

c. Based on type of medication reported in questionnaire.

**Table 5:** Associations Between Obesity and Anxiety and Depression Stratified by Medication Use.

	Medication Users					Medication Non-Users				
	Normal	Overweight		Obese		Normal	Overweight		Obese	
	*N*	*N*	*OR (95% CI)^a^*	*N*	*OR (95% CI)^a^*	*N*	*N*	*OR (95% CI)^a^*	*N*	*OR (95% CI)^a^*
*Daily Antidepressant Use*										
Anxiety										
Yes	101	67	1.20 (0.74–1.94)	46	1.39 (0.79–2.45)	234	148	1.03 (0.82–1.30)	71	0.86 (0.64–1.15)
No	79	44		26		1032	661		385	
Depression										
Yes	161	104	1.23 (0.56–2.66)	73	2.35 (0.77–7.14)	264	152	0.93 (0.74–1.16)	71	0.74 (0.56–0.99)
No	20	11		4		1002	654		382	
*Tricyclic Antidepressant Medication*										
Anxiety										
Yes	46	34	1.18 (0.60–2.33)	29	1.67 (0.79–3.56)	354	205	1.03 (0.83–1.27)	104	0.86 (0.66–1.12)
No	41	25		16		1005	655		380	
Depression										
Yes	71	51	1.17 (0.49–2.81)	40	1.66 (0.60–4.62)	289	181	0.94 (0.77–1.14)	88	0.82 (0.64–1.05)
No	17	10		6		1070	680		395	
*Selective Serotonin Reuptake Inhibitors*										
Anxiety										
Yes	55	26	1.09 (0.52–2.28)	20	1.57 (0.65–3.82)	280	189	1.09 (0.88–1.34)	97	0.96 (0.74–1.24)
No	39	17		9		1072	688		402	
Depression										
Yes	89	40	0.53 (0.15–1.84)	31	2.06 (0.24–17.89)	336	216	1.02 (0.83–1.24)	113	0.92 (0.72–1.17)
No	6	5		1		1016	660		385	

a. Model adjusted for age.

## Discussion

Overall, this study found no association between history of anxiety or depression and risk of being overweight or obese currently, although both were associated with weight gain over time. Taking antidepressants was correlated with obesity among women who reported having been depressed. Results from previous studies have been mixed, with some supporting positive relationships for depression and anxiety with obesity [6]–[26] while others, like this study, have not demonstrated an overall relationship [5], [27]–[30].

The age when women first experienced anxiety or depression had no impact on associations with overweight/obesity. Some longitudinal studies have demonstrated associations between depression diagnosed during adolescence and subsequent obesity that were not replicated here [18]–[21]. However, most have had short follow up periods (often less than 10 years) where the average age of women in our study at the time of obesity assessment greater than in previous work. If adolescent depression only affects obesity in the short term, the difference in time between first having experienced depression and age at obesity assessment might explain why no association between adolescent depression and adult overweight and obesity was seen in this study. Further, some studies of adult depression and obesity have shown positive associations [25], [26], [33], while others have only observed associations between depression and obesity at baseline [9]. Other studies that demonstrate associations between adult depression and obesity have used methods other than BMI (ex. abdominal obesity, weight gain over one year) to characterize obesity, which could explain why we found no associations using BMI [12], [25], [26].

Despite the lack of association with obesity as measured by BMI, this study did demonstrate associations for history of both anxiety and depression with weight gain between age 25 and current weight. Relationships between depression, anxiety and weight gain seen here are consistent with those of some previous studies, where one investigation found an association between childhood depression and weight gain after 20 years in men and women [22], while another found anxiety scores were associated with one-year weight gain in men [26]. A third study found an effect of baseline obesity, where those who were initially heavy and depressed gained more weight than those who were not depressed [33].

However, in assessment of weight at age 25, women were asked to recall weights which could have been 30 – 40 years in the past, producing questions around the accuracy of this data. Several epidemiologic studies have seen strong correlations between measured and self-reported values of past weight [45]–[49], suggesting recalled weights as proxies for measured values in epidemiologic studies. However, when our analysis was stratified at age 50 years, associations were stronger in younger women, such that recall of weight at age 25 among older women may have been less accurate, a factor which should be considered when interpreting associations of anxiety and depression with weight gain in the full study sample.

Consistent with previous work [35], [36], history of antidepressant use, specifically use of TCAs, was associated with obesity among women with a history of depression. The lack of association between SSRIs and overweight and obesity was expected, as this type of medication has not been associated with the same amount of weight gain as classes like TCAs and MAOIs [35], [36]. While significant associations between antidepressant use and obesity were not seen when all participants were included in this analysis, differences in the relationship between depression, anxiety and obesity upon stratification by antidepressant use were suggested, where estimated ORs were generally above 1.0 among those who had taken medication and below 1.0 among those who had not. If true, these observations could help explain the null findings between anxiety, depression and obesity in the overall analysis.

The increased risk of obesity among depressed women taking antidepressants supports a role for these medications as an intermediate between depression and obesity. Alternatively, antidepressant medication could be a proxy for more serious disease, such that severe depression is the true obesity risk factor, however, research around mechanisms through which antidepressant use produces weight gain has suggested that these effects occur independently of both treatment outcome and depression severity [50]. An increased preference for sweet and fatty foods, reductions in resting metabolic rate and changes in neurotransmitter activity, have been suggested as mechanisms through which antidepressant medication might lead to weight gain [50], [51]. Further, individuals taking antidepressants may be more likely to have had their illness diagnosed by a physician to obtain a prescription, thus self-reported history of anxiety or depression in this group may be less prone to misclassification, which could further explain differences in associations when accounting for medication use.

A key strength of this study in the context of existing work was consideration of anxiety and depression, giving this analysis a broader scope compared to those that have focused only on depression [6]–[12], [27], [28]. Consideration of anxiety and depression is important to understanding which disorders specifically are associated with obesity. As well, multiple measures of obesity were used, allowing associations with weight gain but not obesity as assessed by BMI to be demonstrated. This study also evaluated the impact of the age when women first experienced both anxiety and depression in multiple age windows, demonstrating that for weight gain, first experiencing depression/anxiety between the ages of 19 and 30 years appeared to have the greatest impact. To our knowledge no previous studies have specifically conducted this type of analysis, making this an important contribution of our work.

However, this study also has limitations that warrant consideration. Information concerning depression, anxiety, BMI and weight gain was obtained from a single questionnaire and the cross-sectional data precludes establishing temporality, such that the possibility that symptoms of depression or anxiety appeared following obesity development cannot be eliminated. However, information concerning the age when women reported first experiencing each mental illness was available and when those where this was within three years of study participation were excluded, as participants were asked to report their height and weight three years prior to the study, results were unchanged (data not shown).

As well, the use of self-report for assessment of both mental illnesses, and height and weight, may have produced exposure and outcome misclassification. For anxiety and depression, a validated scale was not used in this study and diagnoses were not confirmed by clinical assessment, such that self-reported history of these conditions may have been inaccurate. However, questions concerning each mental illness were based on standard diagnostic categories for psychiatric disorders and previous analysis using these data has demonstrated that the prevalence of the mental illnesses investigated in this study sample are consistent with those of previous research [52], suggesting self-report provided a reasonable assessment of anxiety and depression. Heights and weights used in the calculation of BMI were also self-reported and overestimation of height and underestimation of weight can lead to overall underestimates of BMI in self-reported data [53], which could also have led to misclassification in our study. Memory impairment has also been suggested as a consequence of major depressive disorder [54], [55], although results from one meta-analysis suggested that influences on memory were more common among inpatients [54], where participants in our study were all members of the general population and not receiving care in an inpatient setting. If individuals in our analysis with a history of depression did have memory impairment, leading to poorer recall of exposures such as past weights, this could have produced a differential misclassification in our data and the direction of this bias would be difficult to predict.

History of antidepressant medication use was also self-reported by participants, which could raise concerns regarding misclassification or recall bias. However, given that the data used for this study is taken from controls in a breast cancer case-control study [39], [40], it seems unlikely that participants would report antidepressant use differently based on obesity status, reducing the likelihood that recall bias would have a major impact on observed results. Further, the study questionnaire listed multiple medication types in addition to antidepressants [39], reducing the emphasis of the study on antidepressant use specifically.

While this study systematically considered the influence of most potential confounders of mental illness-obesity relationships, total caloric intake (a determinant of obesity) was not available and thus there is potential for confounding of effect estimates. However, overeating has also been suggested as a mechanism through which mental illnesses like depression might influence obesity [5], such that it may be part of the causal pathway and not a true confounder. Finally, while the ability to consider relationships of anxiety and depression with obesity stratified by antidepressant medication use is a strength of this study, the wide confidence intervals around these estimates indicate differences in estimated ORs between the medication user and non-user groups should be interpreted cautiously. To our knowledge no previous studies have examined associations between anxiety or depression and obesity in this way so future research will help provide context to our findings.

In summary, this study did not observe a relationship of anxiety or depression with overweight and obesity, but did find an association with weight gain since age 25. Consideration of anxiety and depression makes an important contribution to the existing literature which has focused mainly on depression. Future prospective studies with assessment of obesity at multiple time points will provide further insight into the impact of depression and anxiety on weight gain observed here, with a greater ability to examine the influence of temporality on these relationships.

## Supporting Information

Table S1 **Associations Between Anxiety, Depression and Weight Gain Stratified by Current Age.**
(DOCX)
