# Supplementary material for: Associations between Anxiety, Depression, Antidepressant Medication, Obesity and Weight Gain among Canadian Women
Source: PLoS One. 2014 Jun 16;9(6):e99780. doi: 10.1371/journal.pone.0099780 (PMC4059657; doi:10.1371/journal.pone.0099780)
Supplement: Table S1 — Associations Between Anxiety, Depression and Weight Gain Stratified by Current Age. (DOCX) [file pone.0099780.s001.docx]

**Table S1: Associations Between Anxiety, Depression and Weight Gain Stratified by Current Age**

| **Mental Illness** | **AGE < 50** | | | | | **AGE ≥ 50** | | | | |
| --- | --- | --- | --- | --- | --- | --- | --- | --- | --- | --- |
|  | **0 – 4 kg (n = 381)** | **5 – 19 kg (n = 374)** | | **>20 kg (n = 69)** | | **0 – 4 kg (n = 631)** | **5 – 19 kg (n =1119)** | | **>20 kg (n =343)** | |
|  | *N* | *N* | *OR*  *(95% CI)^a^* | *N* | *OR*  *(95% CI)^a^* | *N* | *N* | *OR*  *(95% CI)^a^* | *N* | *OR*  *(95% CI)^a^* |
| **Ever Diagnosis:** |  |  |  |  |  |  |  |  |  |  |
| Anxiety |  |  |  |  |  |  |  |  |  |  |
| Yes | 90 | 103 | 1.14 (0.81 – 1.58) | 26 | 1.78 (1.02 – 3.10) | 119 | 254 | 1.28 (1.00 – 1.63) | 77 | 1.27 (0.92 – 1.76) |
| No | 290 | 265 |  | 41 |  | 503 | 843 |  | 259 |  |
|  |  |  |  |  |  |  |  |  |  |  |
| Depression |  |  |  |  |  |  |  |  |  |  |
| Yes | 115 | 125 | 1.10 (0.80 – 1.50) | 34 | 2.12 (1.25 – 3.60) | 173 | 281 | 0.89 (0.71 – 1.11) | 95 | 1.04 (0.77 – 1.39) |
| No | 263 | 246 |  | 34 |  | 447 | 820 |  | 239 |  |
|  |  |  |  |  |  |  |  |  |  |  |
| Depression/Anxiety |  |  |  |  |  |  |  |  |  |  |
| Yes | 146 | 158 | 1.10 (0.81 – 1.48) | 39 | 1.89 (1.11 – 3.19) | 209 | 379 | 1.04 (0.84 – 1.28) | 118 | 1.07 (0.81 – 1.42) |
| No | 234 | 214 |  | 30 |  | 418 | 732 |  | 222 |  |

1. Model adjusted for age.
